# The perspectives of a broader user population on the dents AR Augmented Reality application for children’s oral health promotion based on gender, age, and grade in rural areas: a cross sectional study using the modified system usability scale

**DOI:** 10.1186/s12903-026-07971-x

**Published:** 2026-03-04

**Authors:** Anne Agustina Suwargiani, Ardena Maulidia Hamdani, Fidya Meditia Putri, Yolanda Yolanda, Sri Susilawati, Netty Suryanti, Mira Suryani, Niekla Survia Andiesta, Dudi Aripin

**Affiliations:** 1 Dental Public Health Department, Faculty of Dentistry Universitas Padjadjaran, Bandung, Indonesia; 2 Conservative Dentistry Department, Faculty of Dentistry Universitas Padjadjaran, Bandung, Indonesia; 3 Computer Science Department, Faculty of Mathematics and Natural Sciences Universitas Padjadjaran, Bandung, Indonesia; 4 Division of Children and Community Oral Health, School of Dentistry International Medical University, Kuala Lumpur, Malaysia

**Keywords:** Augmented reality, Usability, Children, Oral health, Promotion, Usability scale

## Abstract

**Introduction:**

The augmented reality (AR) application Dents AR was developed to align with technological and informational advancements in a field that requires a valid framework to evaluate usability aspects effectively. This study aimed to analyse the perspectives of children in rural areas on the Dents AR augmented reality application based on gender, age, and grade using the Modified System Usability Scale (mSUS).

**Method:**

A cross sectional design was implemented, involving 380 children from three elementary schools. A purposive sampling procedure was applied, and the sample size was determined using the population proportion formula for a known population size with 5% precision, resulting in a final sample of 195 children. Data collection utilized the Dents AR application from the Augmented Reality Activity Book: Dental Caries Progress And How To Prevent It and the application was launched by scanning the barcode provided in the book. Data was administered through online questionnaires. Data were analyzed using the T test and One way Anova tests.

**Results:**

The mSUS score range and grade distribution for the Dents AR application from grade F (poor) to grade A+ (excellent). Approximately 46.6% of responses fell within the “F&D” grade category or poor adjective category and approximately 53.3% of responses fell within the “C until A+” grade category or the OK to excellent adjective rating. The comparison of mSUS scores based on participant characteristics revealed that the p-values for gender (*p* = 0.245), age (*p* = 0.591) and grade level (*p* = 0.720) were all greater than 0.05, indicating no statistically significant differences in mSUS scores by gender, age or grade level.

**Conclusion:**

The perspectives of a broader user population on the dents ar augmented reality application for children’s oral health promotion based on gender, age, and grade in rural areas: a cross sectional study using the modified system usability scale shows that the Dents AR application had values from grade F (poor) to grade A+ (excellent). Approximately more responses fell within the “F&D” grade category or poor adjective category compared to responses that fell within the “C until A+” grade category or the OK to excellent adjective rating and perspectives across gender, age, and grade revealed no usability differences.

## Background

Based on our previous research on caries in rural areas with limited digital literacy, we found that the International Caries Detection and Assessment System (ICDAS) evaluation revealed a caries prevalence of 91.4% for permanent teeth. ICDAS codes range from 1 to 5, with code 5 indicating the most severe stage of caries. [1] These conditions require intervention to promote oral health, with augmented reality (AR) being one potential solution to enhance knowledge, attitudes, and practices through the direct visualization of clinical information.

In dentistry, AR enhances clinical practice by enabling the direct overlay of clinical information on the patient, thereby merging real and virtual environments. Its primary application in dentistry is to deliver digital information that enriches clinical experiences, facilitating more effective communication between patients and dentists through videos, images, and three dimensional models. [2–4].

The utility of AR systems in dental education is comparable to simulation training that utilizes dental models, anatomical models, phantom heads, and actors. Simulation training provides valuable experience and has long been an established method for teaching dentistry. In contrast, virtual reality (VR) and AR systems usher in a new era of realism, significantly enhancing the learning experience. AR technology allows user interaction with integrated images of the patient’s teeth and anatomical structures. [2].

AR is considered one of the most effective tools in educational technology due to its ability to enhance learning outcomes [5] and significantly improve oral health knowledge, attitudes, and self-efficacy, ultimately contributing to better overall health outcomes. [6,7] Furthermore, AR can serve as a useful supportive tool in clinical practice. [5] Real time observation of both visualizations and actual teeth, along with customized and comprehensive lessons, enables highly accurate evaluations, making it an effective and standardized tool. [8].

Based on the ICDAS framework, this application features eight AR scenes accessible to users. These scenes begin with an introduction to the oral cavity tailored to the user’s age, followed by an overview of dental anatomy and dental caries, including their development. Furthermore, visual information about caries prevention is provided in a separate menu and is comprehensively presented in an activity book, which also serves as the AR marker. The target audience for this AR application includes individuals of all ages, with a focus on younger users accompanied by their parents. [8].

Theories and usability frameworks were considered when designing applications for children, particularly to support the integration of AR with recognized usability principles. First, our reference is ISO 9241 − 11 [9], which defines usability in terms of effectiveness, efficiency, and satisfaction in a specified context. These three aspects were reflected in both the design goals of our AR prototype and our evaluation method, which assessed how easily and enjoyably children could complete learning tasks using the AR system. [10].

Second, we incorporated Nielsen’s heuristics [11,12] particularly the match between system and the real world, consistency and standards, and visibility of system status, into the design process. These heuristics were adapted to suit a younger audience by simplifying interactions, using child friendly visual metaphors, and providing clear feedback through multimodal cues.[10,13].

According to the literature, current developments are insufficient for a full validation process. However, an independent source of specialized software for AR appears promising in supporting routine procedures, complex and specialized interventions, and educational activities. [12,14] Therefore, the evaluation of validity remains essential. Various aspects can be evaluated, including interaction techniques, user efficiency and accuracy. [2,10,15].

Previous studies indicate that certain individuals, particularly school aged children, find it challenging to use AR. This issue is considered a major obstacle to the implementation of AR in education. [16,17] As such, usability evaluation is crucial to assess various aspects of the AR usability, including effectiveness, efficiency, and user satisfaction. [18,19] Furthermore, it is important to evaluate the usability of this AR application in supporting children’s cognitive development. [10].

This study utilized the mSUS questionnaire to assess various aspects of usability based on users’ subjective evaluations. [18,20–23] This scale is among the most widely used questionnaire based methods for evaluating usability. It is commonly used for its ability to provide insight into users’ perceptions. [19] Furthermore, the mSUS method serves as a performance indicator to assess the long term usability of a digital product. [6,16,24].

Although the mSUS was originally developed for adults, recent studies have shown that it can be adapted for children with appropriate guidance and simplified language. In this study, we administered the mSUS in an interview style format to ensure comprehension. This approach has been validated in prior usability studies involving children aged 8–12 years. [10,15,16,24].

In our study, the sample size was intentionally large because the objective was not merely to identify usability issues through exploratory methods such as think aloud protocols, but rather to evaluate the perceived usability of the AR application from the perspectives of a broader user population (children). Accordingly, the study adopted a summative usability evaluation approach, aiming to obtain findings regarding overall user perceptions of the system’s usability. [25].

The novelty of this study lies in the fact that the Dents AR application is a newly developed tool that has not previously been evaluated. Furthermore, the mSUS evaluation of the Dents AR application represents a pioneering effort in this field. This study aimed to analyse the perspectives of a broader user population on the dents ar augmented reality application for children’s oral health promotion based on gender, age, and grade in rural areas.

## Methods

Cross sectional analytic studies are a type of research designed to describe a phenomenon or explore relationships between variables at a specific point in time. [26,27] These studies can be thought of as a “snapshot” of the frequency and characteristics of a condition within a population at a given moment. [28].

The research population consisted of 380 children from three elementary schools. Inclusion criteria for the study population included elementary school aged children, willingness to participate as research respondents, parental consent, and interest in learning about the Dents AR application. Exclusion criteria included children who were unwell, had visual impairments or other vision problems, or did not complete all stages of the research.

The sample for this study was drawn from elementary school children using a purposive sampling technique. [29] The sample size was calculated using the formula for estimating a population proportion with a known population size n = N/(1 + Nd^2^). [24] The final sample of 195 children was drawn from three elementary schools: Puteran 1, Puteran 2, and Sirnagalih Elementary Schools.

We included a relatively large sample size (*n* = 195 analyzed from a population of 380) to ensure statistical robustness, adequate representation across varying age groups, usage contexts, and levels of digital familiarity among children, and to allow meaningful subgroup comparisons (e.g. by age, gender, or prior experience with AR or digital tools).

The variables in this research included the Dents AR augmented reality application and the mSUS. Dents AR is a product designed for oral health promotion that uses ISO 9241-11 and incorporates Nielsen’s usability heuristics as theoretical frameworks. The Dents AR system combines a mobile application system with an activity book.

**Fig. 1:**
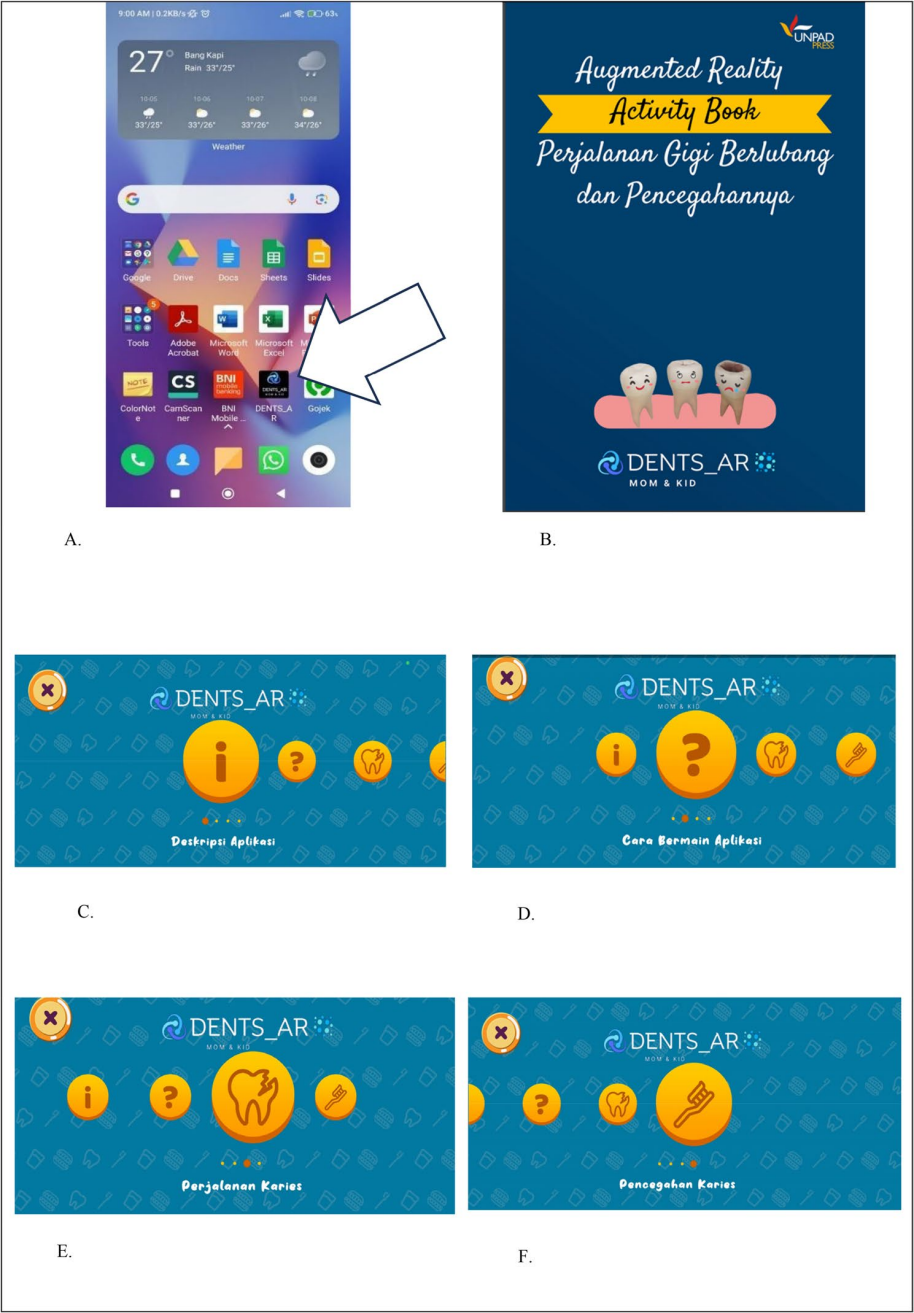
Dents AR is an augmented reality system that integrates the Dents AR mobile application via a barcode in the Augmented Reality Activity Book: Dental Caries Progress and How To Prevent It [8]. **A** Mobile Application Dents-AR installed after scanning barcode on page 2 of the book at Figure 1B. **B** The title of book is Augmented Reality Activity Book: Dental Caries Progress and How To Prevent It. **C** Click  on the application to view the description of how to use the Dents AR application. **D** Click to learn how to play the Dents AR application. **E** Click  to Learn about to learn about caries development in Dents AR. **F** Click  on the application to learn about the prevention of caries in Dents AR

To use this application, users must first download the Dents AR application from the Augmented Reality Activity Book: Dental Caries Progress and How To Prevent It and launch the application by scanning the barcode provided on page 2 of the book (Fig. 1B). The Book is available at https://bookstore.unpad.ac.id/product/augmented-reality-activity-book-perjalanan-gigi-berlubang-dan-pencegahannya/. Figure 1. Dents AR is an augmented reality system embedded via a barcode in the Dents AR mobile application and used with the Dents AR activity book. [8].

The application runs on Android 11 or higher with One UI 3.1 or above, and utilizes the rear camera to scan markers, with a main camera specification of 48MP, f/2.0, wide lens. Regarding lighting conditions, the acceptable range is fairly broad, with optimal performance observed under uniform illumination, such as in indoor environments.

The mSUS measures the usability of a system through a questionnaire designed to capture users’ subjective perceptions; in this study, it was used to assess the experiences of children using the Dents AR application. [11,20,30].

The mSUS instrument consists of 10 items divided into two subscales: usability, comprising items 1, 2, 3, 5, 6, 7, 8, and 9; and learnability, comprising items 4 and 10. Positive statements include items 1, 3, 5, 7, and 9, while items 2, 4, 6, 8, and 10 are negatively worded. Respondents were asked to assess the usability of a product using a 5 point Likert scale, where a score of 1 indicates “strongly disagree” and a score of 5 indicates “strongly agree”.[20, 30]

**Table 1:** English translation of questionnaire items based on an adapted Indonesian child version of the mSUS for child validated usability scale

No	Question Items in English
1	I think I would like to use this system again (Positive statement)
2	I thought this system was complicated to use. (Negative statement)
3	I thought this system was easy to use. (Positive statement)
4	I would need the support of someone else or a technician to use this system. (Negative statement)
5	I thought the various features in this system worked as expected. (Positive statement)
6	I thought there were many things that were inconsistent (incompatible) on this system (Negative statement)
7	I thought other people would learn how to use the system quickly. (Positive statement)
8	I thought this system was confusing to use. (Negative statement)
9	I thought there were no difficulties in using this system. (Positive statement)
10	I need to get familiar with it before I can use this system. (Negative statement)

These items are translated from the Indonesian adaptation of the mSUS used in this study and may differ slightly from the original version developed by Brooke (1996)

Table 1. English translation of questionnaire items based on an adapted Indonesian child version of the mSUS for child validated usability scale. The child validated usability scale was developed through translation and back translation by two English language experts. Content validity was assessed by three experts in the field of public oral health and information technology, yielding a Content Validity Index (CVI) of 0.80, indicating acceptable item validity. The CVI was calculated based on research related to the validity of assessment instruments. [25].

Face validity was conducted through expert judgement on the questionnaire items by three professionals specializing in public oral health and information technology. The resulting FVI demonstrated agreement on the adapted version, with a value of 0.80, confirming that the adapted questionnaire items were considered valid. Face validity testing was also performed using the adapted questionnaire version yielding the same FVI value. [26].

**Table 2:** Factor loadings from exploratory factor analysis (varimax method)

Item		Loading factor		
No	Formulation	1 (Complexity)	2 (Learnability)	3 (Familiarity)
2	I thought this system was complicated to use	0.61		
5	I thought the various features in this system worked as expected	0.74		
6	I thought there were many things that were inconsistent (incompatible) on this system	0.79		
8	I thought this system was confusing to use	0.71		
1	I think I would like to use this system again		0.70	
3	I thought this system was easy to use		0.75	
7	I thought other people would learn how to use the system quickly		0.66	
9	I thought there were no difficulties in using this system		0.54	
4	I would need the support of someone else or a technician to use this system			0.80
10	I need to get familiar with it before I can use this system			0.83

Construct validity of the mSUS instrument was analysed using exploratory factor analysis (EFA) see Table 2. Based on EFA, the Indonesian version of mSUS shows three factors that are slightly different from the English version. Within the usability subscale, the first factor represents complexity, encompassing items 2, 5, 6, and 8, which capture children’s perceptions that the system is complicated, confusing, or inconsistent. Higher scores on this factor indicate lower perceived usability due to system complexity. The second factor represents learnability, including items 1, 3, 7, and 9, reflecting ease of use, intuitiveness, and learnability, as well as users’ willingness to use it again and eir perception of how quickly others could learn the system.

The learnability subscale remained constant, measured by items 4 and 10, which form the construct termed familiarity, that represents the extent to which children feel they require assistance or prior experience to use the system effectively. The factor loadings indicated that no items required removal: all factor loadings exceeded the standard exclusion criterion (a loading < 0.30 on either factor and a loading difference < 0.15 between factors).

The Kaiser-Meyer-Olkin measure of 0.65 confirmed sample adequacy. Bartlett’s test for sphericity was significant ($\:{\chi\:}^{2\:}=302.41,\:df=45$, *p*<0.001), indicating that the inter item correlations were adequate. This analysis accounted for a cumulative explained variance of 55.03% across the two factors.

**Fig. 2:**
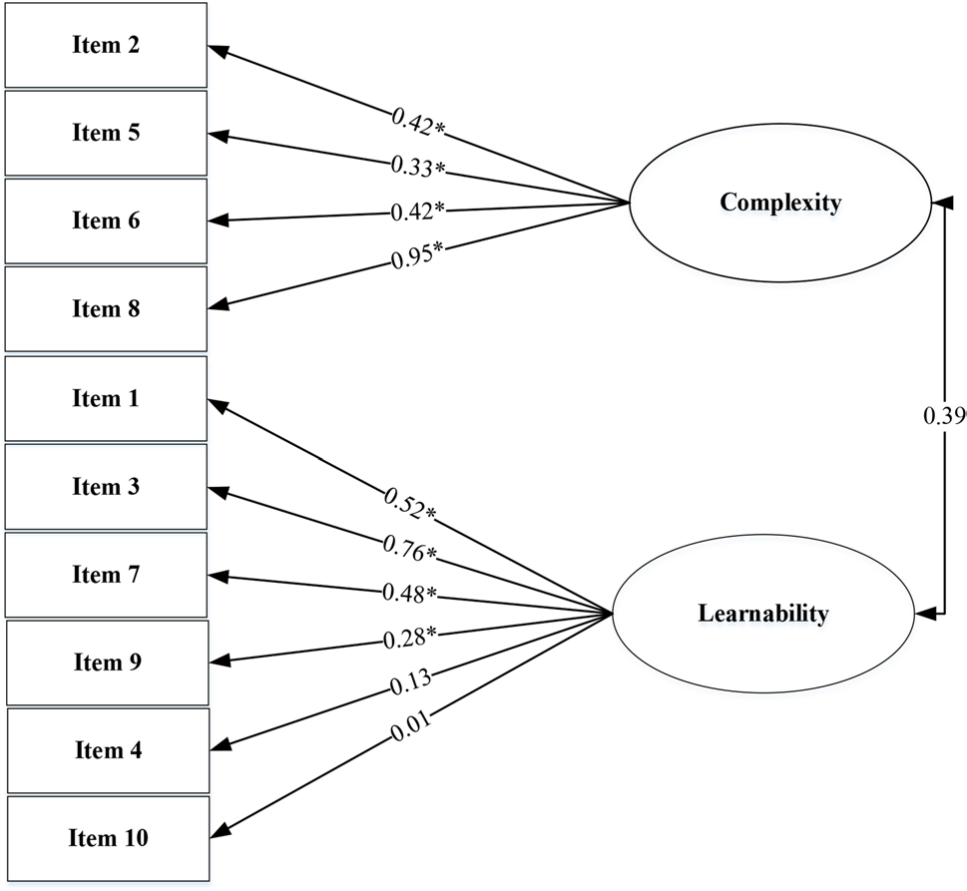
The two factor model using 10 items based on the findings of EFA

**Fig. 3:**
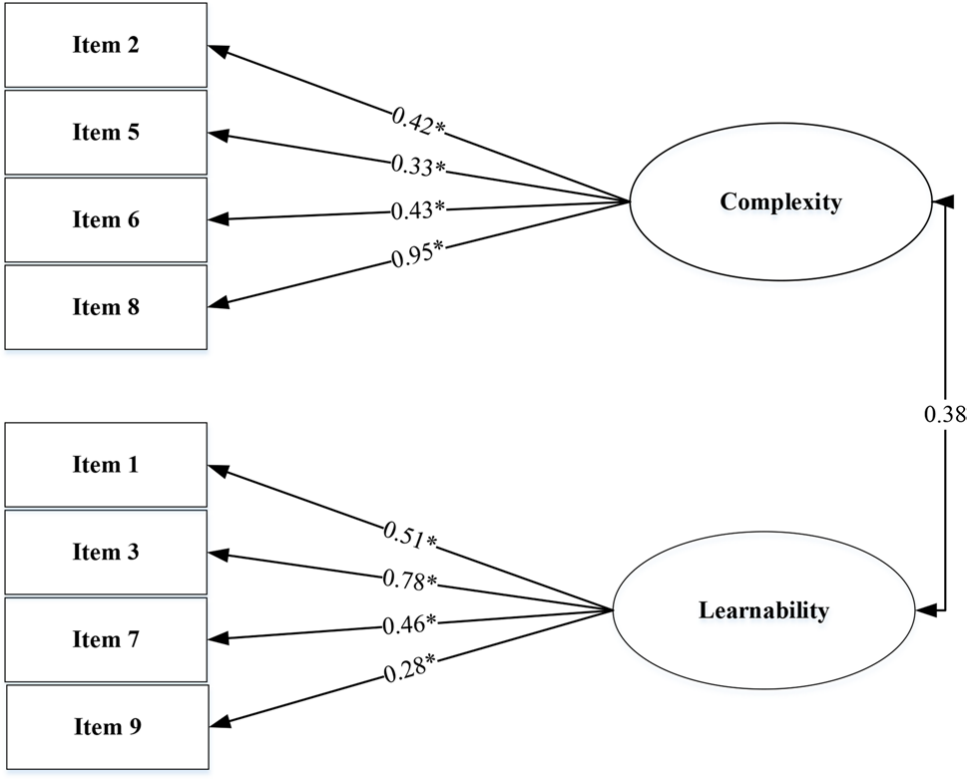
Two factor model derived from exploratory factor analysis (EFA) using 8 items, after the exclusion of items 4 and 10

The internal structure validity of mSUS was evaluated by the confirmatory factor analysis (CFA) using LISREL 8.8 and the factor model being analysed was based on the finding of exploratory factor analysis (EFA) (Figs. 2 and 3).

Figure 2 shows the results of the exploratory factor analysis (EFA), which identified two factors. The first model consisted of 10 items loading onto two factors (usability and learnability). However, items 4 and 10 showed factor loadings that were below the acceptable threshold. Therefore, EFA was conducted again using 8 items after excluding items 4 and 10, resulting in acceptable factor loadings for all remaining items (Fig. 3).

Model fit was evaluated using the comparative fit index (CFI), Tucker-Lewis Index (TLI), and root mean squared error of approximation (RMSEA). CFI, and TLI index values range from 0 to 1.0, with values above 0.90 indicating a good fit to the data. Smaller values of RMSEA indicate a better fit, with values < 0.10 implying a good fit, and values < 0.05 considered a very good fit. [31].

Internal consistency was assessed using Cronbach’s Alpha, [26] based on responses from 34 children to the 10 item mSUS questionnaire. The resulting Cronbach’s Alpha value of 0.776 exceeded the 0.70 threshold, confirming that the questionnaire is reliable and suitable for usability evaluation.

For scoring, contributions from positively worded items were calculated by subtracting 1 from each rating, while scores for negatively worded items were obtained by subtracting the rating from 5. The total mSUS score was then calculated by summing all item scores and multiplying the result by 2.5, producing a final score between 0 and 100 (Table 3). Although the mSUS scores are presented on a 100 point scale, these values do not represent percentages. [27].

**Table 3:** mSUS Grade Scale Classification and Adjective Ratings [21]

Grades	mSUS Scales	Adjective Rating
A+	84.1–100	Best Imaginable
A	80.8–84.0	Excellent
A-	78.9–80.7	Good
B+	77.2–78.8	
B	74.1–77.1	
B-	72.6–74.0	
C+	71.1–72.5	
C	65.0–71.0	OK
C-	62.7–64.9	
D	51.7–62.6	
F	25.1–51.6	Poor
F	0–25	Worst Imaginable

Data collection for the Dents AR augmented reality application, used as an oral health promotion tool, was conducted using an online questionnaire system based on an Indonesian adaptation of the mSUS. [10] Scores were analyzed using frequency distributions and mSUS grade scale classification. [21] The average mSUS score was calculated using mean x̅ = Σx/n. [29]

The formula used to calculate the mean score from a usability testing system. The components of Eq. 1 are as follows: x̅ = Average Score; ∑x = Total mSUS Score; and n=number of respondents. Data were analyzed using frequency distribution analysis of the mean mSUS score, with mSUS categories presented on a 100 point scale. The T test and One way anova tests were used to compare usability scores across gender, age and grade/class.

All participants provided written informed consent prior to data collection. The study was approved by the Institutional Review Board (Health Research Ethics Committee) of the Faculty of Medicine, Universitas Padjadjaran, Indonesia. Ethical clearance was granted under approval number 1174/UN6.KEP/EC/2022, November, 21 2022. This study was funded by an internal research grant from Universitas Padjadjaran, Grant Number 1768/UN6.3.1/PT.00/2024. The authors declare no conflict of interest, including in relation to the funding received.

A pilot study was conducted to evaluate the usability and feasibility of the Dents AR application. Ten participants (schoolchildren) tested the system and completed a usability questionnaire. Descriptive and qualitative feedback were analyzed, and the results showed that all participants reported interaction flow, visual clarity, user comfort, linguistic choices and learnability to be very good and not overly complex.

## Results

The results of the evaluation of the Dents AR augmented reality application as a digital oral health promotion tool, along with the findings from the mSUS, were presented in Tables 4 and 5, and 6.

**Table 4:** Distribution of children’s responses to mSUS items for the Dents AR application

No	Modified System Usability Scale	Strongly disagree		Disagree		Neutral		Agree		Strongly agree	
		f	%	f	%	f	%	f	%	f	%
	Complexity										
Q2	This system was complicated to use. (Negative statement)	19	9.7	59	30.3	41	21.0	58	29.7	18	9.2
Q5	The various features in this system worked as expected (Positive statement)	37	19.0	73	37.4	45	23.1	29	14.9	11	5.6
Q6	There were many things that were inconsistent with this system. (Negative statement)	8	4.1	32	16.4	41	21.0	69	35.4	45	23.1
Q8	This system was confusing to use. (Negative statement)	11	5.6	44	22.6	36	18.5	73	37.4	31	15.9
	Learnability										
Q1	I would like to use this system again. (Positive statement)	4	2.1	40	20.5	38	19.5	76	39.0	37	19.0
Q3	This system was easy to use. (Positive statement)	3	1.5	25	12.8	36	18.5	94	48.2	37	19.0
Q7	Other people would learn how to use the system quickly. (Positive statement)	9	4.6	26	13.3	35	17.9	85	43.6	40	20.5
Q9	There were no difficulties in using this system. (Positive statement)	10	5.1	47	24.1	35	17.9	87	44.6	16	8.2

Table 4 presents the distribution of mSUS responses for the Dents AR application. The responses of each subscale were varied widely, ranging from “strongly disagree” to “strongly agree”, indicating diverse perceptions of usability among users.

Table 5 shows the mSUS score range and grade distribution for the Dents AR application from grade F (poor) to grade A+ (excellent). Approximately 46.6% of responses fell within the “F&D” grade category or poor adjective category and approximately 53.3% of responses fell within the “C until A+” grade category or the OK to excellent adjective rating.

**Table 5:** Frequency distribution of mSUS grades for the Dents AR application (*n* = 195)

Grade	Adjective rating		Frequency		Percent		
F		Poor		17		8.7	
D		Poor		74		37.9	
Total				**91**		**46.6**	
C		OK		64		32.8	
Total				**64**		**32.8**	
C+		Good		13		6.7	
B		Good		9		4.6	
B+		Good		4		2.1	
A-		Good		2		1.0	
Total				**28**		**14.4**	
A		Excellent		9		4.6	
A+		Excellent		3		1.5	
Total				**12**		**6.1**	

**Fig. 4:**
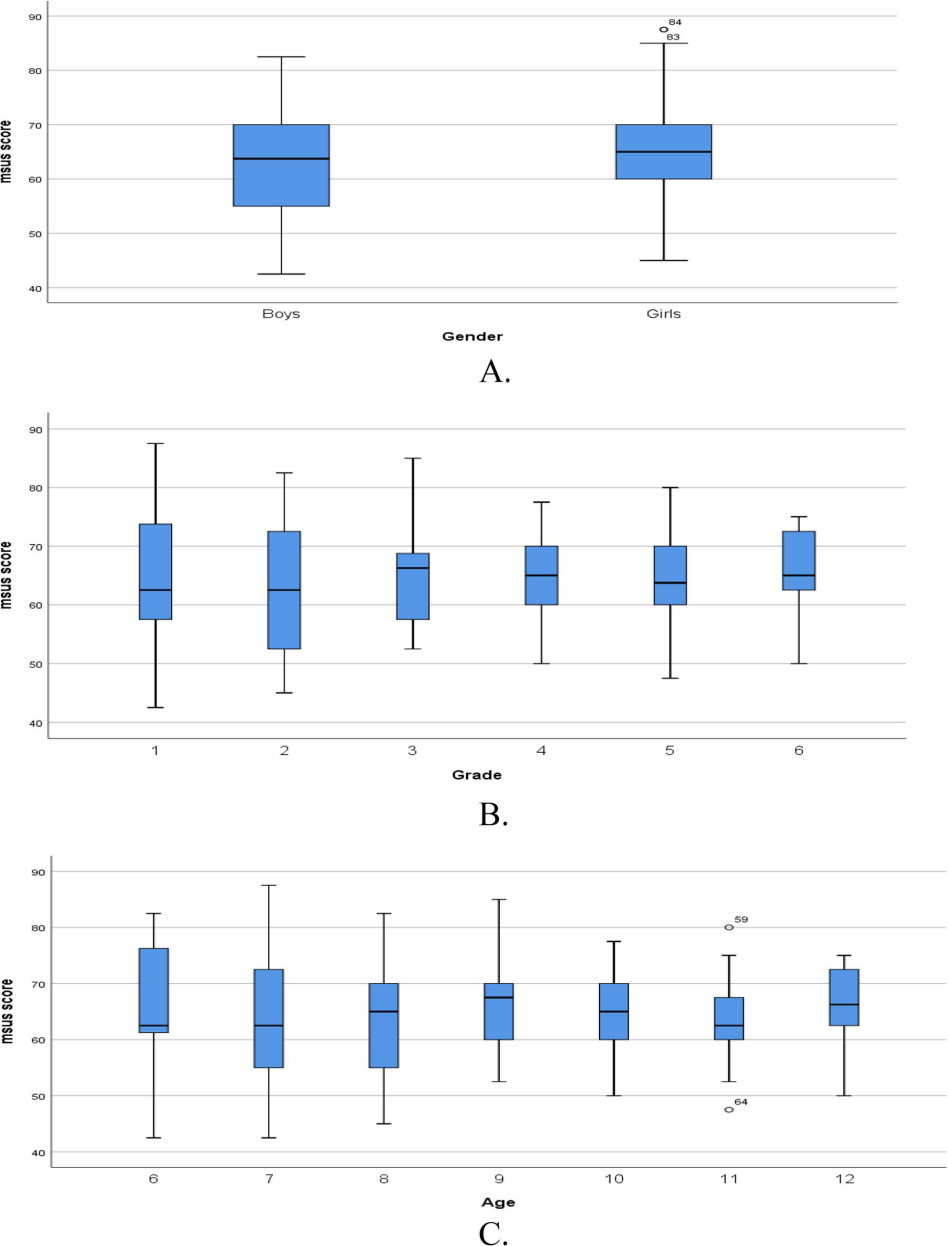
Boxplots of mSUS score distribution by (**A**) gender, (**B**) grade level, and (**C**) age among elementary school children. **A** mSUS score distribution by gender **B** mSUS score distribution by grade **C** mSUS score distribution by age

Figure 4 shows the mSUS score distribution by (A) gender showing that mSUS scores have median values between 60 and 70. The distribution by (B) grade level also shows median mSUS scores between 60 and 70 and the distribution by (C) age among elementary school children shows a similar score distribution, with median values between 60 and 70.

Table 6 summarizes respondents’ characteristics by gender, age and grade level. The gender distribution indicated that there were more female than male participants. The participants’ ages ranged from 6 to 12 years, with the largest group being 10 year olds. The table also presents the grade level distribution, showing that most participants were from grade 4, followed by grades 1 and 6.

**Table 6:** Participant characteristics and comparison of mean mSUS scores (*n* = 195)

Characteristics	*n*	%	Mean (SD) mSUS score	Statistical test	*p*-value
Gender: Male Female Age (Years): 6 7 8 9 10 11 12 Mean (SD): 10 (1.75) Grades (Class): 1 2 3 4 5 6	78 117 7 29 37 20 46 30 26 32 37 20 48 28 30	40.0 60.0 3.6 14.9 19.0 10.3 23.6 15.4 13.3 16.4 19.0 10.3 24.6 14.4 15.4	63.46 (10.06) 65.04 (8.69) 66.07 (13.53) 63.62 (12.39) 63.65 (11.71) 66.25 (7.93) 63.97 (6.96) 63.58 (7.36) 66.25 (6.76) 64.53 (12.21) 63.65 (12.80) 64.25 (7.61) 64.27 (6.78) 64.38 (7.19) 64.41 (9.27)	t(df = 193)= -1.17 F(6.188) = 0.47 F(5.189) = 0.15	0.16 0.56 0.76

As shown in Table 6, the comparison of mSUS scores based on participant characteristics revealed that the p-values for gender, age, and grade level were all greater than 0.05. These results indicate that there were no statistically significant differences in mSUS scores by gender, age or grade level, suggesting consistent perceptions of usability for the Dents AR application across these demographic variables.

## Discussion

The number of respondents varied by age and grade level, likely reflecting differing levels of willingness to complete all stages of the research, as well as factors related to parental consent (Table 6). These differences are consistent with previous findings suggesting that AR applications provide short term immersive experiences, regardless of participants’ age and gender. [28] Based on the authors’ observations and interviews, this was the first AR experience for grade 3 students at the elementary schools in Puteran Village.

This research builds upon the findings of previous studies conducted by Suwargiani et al. at the same site, which highlighted the need for health promotion interventions and caries prevention programs to mitigate dental caries progression. [32] Consequently, the Dents AR application was developed as a digital oral health promotion tool. This study evaluated its usability among elementary school aged children in Puteran Village, a rural area in Tasikmalaya, West Java, Indonesia, using the mSUS instrument.

Elementary school aged children from three elementary schools, Puteran 1, Puteran 2, and Sirnagalih, were included. These schools were intentionally selected as sampling locations because they appropriately represent rural educational settings that are relevant to the aims of this study. These schools are located in the village characterized by low population density, limited infrastructure development, and livelihoods that are predominantly based on agriculture and informal economic activities. These characteristics are key indicators of rural areas. [33,34].

These schools provided environmental and cultural homogeneity, which helps reduce extraneous variability that could arise from differing regional conditions. Typical rural characteristics associated with these schools include limited access to learning facilities, constrained educational resources, and minimal exposure to supplementary academic programs, and the student population largely comes from families with similar socio-economic backgrounds. [33,34].

The comparison of mSUS scores by participant characteristics (Table 6) showed no significant differences between male and female participants, and across different age groups or grade levels. This finding is supported by Fig. 4, which shows mSUS scores at similar levels based on gender, age and grade levels. These findings suggest that perceived usability and user experience of the Dents AR application comprising both the mobile app and the accompanying activity book did not differ significantly between boys and girls, across various age levels in children aged 6–12 years and at different grade levels.

These results differ from those reported by Huang and Mou, who found that, in a gender context, the usability of existing online websites based on user perceptions and performance showed that women had greater usability needs than men. [39] However, they are consistent with prior research on gender differences in game based learning, which reported similar levels of interest and engagement among male and female students. [35].

This result suggests that the Dents AR application is well received by both boys and girls across all elementary grade levels, demonstrating usability that is acceptable for young learners. This result may occur because the Dents AR application was designed using a child-centered design approach, which places children as the primary users and considers cognitive development, motor skills, emotional aspects, and interaction capabilities. [36] A child-centered design approach produces a better user experience, increases engagement, and supports children’s learning process. [37].

Table 4 shows that responses varied widely, ranging from “strongly disagree” to “strongly agree”, reflecting diverse user perceptions of usability among respondents. These findings are consistent with meta analytical field studies reporting that user perceptions of digital application usability often vary across individuals, reflecting differences in background and experience. These findings correspond with the demographic characteristics of the respondents in Table 6, which show variations in age, grade level, and limited exposure to AR based digital literacy. [1,32].

In addition, elementary school-aged children, typically between the ages of 6 and 12, experience significant cognitive, social, and emotional development. According to Piaget’s theory of cognitive development, children at this age are in the concrete operational stage, a phase in which they begin to think logically, classify objects, and understand cause-and-effect relationships, but still rely on concrete and visual experiences and representations. [38].

According to mSUS response distribution (Table 4), respondents expressed positive responses to Question 1 (I think I would like to use this system again). These results are consistent with Lupton’s findings [25], which suggest that young individuals appreciate the availability of online information, opportunities to learn more about their bodies and conditions, and tools to improve health. Observational data further indicated that participants were enthusiastic about using the application, though each was limited to a single session due to time constraints.

Previous research by Korosidou et al. demonstrated that AR based learning creates immersive and engaging experiences that are both pleasant and conducive to enhancing learning ability. [39] The willingness to utilize this Dents AR system presents significant opportunities for creative and innovative thinking, particularly when the younger generation is encouraged to participate. [32] The usability results highlight opportunities to enhance learning ability, creativity and innovative perspectives related to oral health and overall health improvement.

Based on the findings in Table 4, responses to the positive statement in Question 3 (I thought this system was easy to use) further support the perception of usability. This ease of use may stem from clear, short term interactive features within the system. This finding is supported by Roumba and Nicolaidou, who reported that short term interactions with AR or books may motivate younger individuals to read, thereby improving their understanding of these media. [40] The results of this study suggest that, through the user friendly Dents AR oral health promotion tool, students gained a valuable learning experience.

In contrast, these findings differ from those of Mune et al., who reported the potential for visual fatigue associated with AR use—an issue that must be addressed to enhance its successful integration across all age groups. [41] This discrepancy may be due to the concise and linear structure of the Dents AR application, which is presented sequentially, starting with an overview of the application, followed by usage instructions and concluding with explanations of caries development and prevention.

Respondents’ answers to Question 9 (I thought there were no difficulties in using this system) showed both agreement and disagreement (Table 4). However, these responses contrast with Al Kalbani’s study, which reported common challenges and obstacles associated with immersive technology. [42] This difference may be due to the Dents AR application being developed as an oral health promotion product based on user needs specifically, the need to enhance users’ knowledge about dental caries. [8,32].

As users, the majority of respondents expressed disagreement with Question 5 (I thought the various features in this system worked as expected) and Question 7 (I thought other people would learn how to use this system quickly) (Table 4). These responses align with a study conducted by Oke et al., which stated that a primary barrier to using AR was the first time experience with an AR application, particularly the challenges users may have encountered during use, their lack of technological knowledge, and difficulties in setting up AR technology. [43] Furthermore, this is consistent with previous studies on barriers to system usage. This research was conducted in a rural area where the use of smartphones and tablets for AR among children remains limited. Consequently, psychological barriers and obstacles related to mobile technology usage persist in the context of AR.

In addition, Table 4 indicates disagreement reflected by frequencies exceeding 40 respondents for the negative statements from the following questions: Q2 (I thought this system was complicated to use), Q6 (I thought there were many things inconsistent with this system), and Q8 (I thought this system was confusing to use). These findings align with Sugiono’s study [44], whose SWOT analysis also indicated that public acceptance of AR remains limited.

Some of the challenges in developing AR include a lack of acceptance among the general public. Currently, this technology is not fully accepted by the community, as many individuals find it difficult to use. Additionally, AR requires certain technological specifications to function effectively. [44] These challenges highlight the difficulties in promoting the Dents AR application as an oral health promotion tool among elementary school children, particularly in rural areas where residents may be less familiar with technological advancements, and smartphone usage remains low.

Table 5 shows the mSUS score range and grade distribution for the Dents AR application from grade F (poor) to grade A+ (excellent). Approximately 46.6% of responses fell within the “F&D” grade category or poor adjective category and approximately 53.3% of responses fell within the “C to A+” grade category or the OK to excellent adjective rating (see Table 5). These findings contrast with Najaf et al., who found that mSUS scores for usability per user ranged from 72.5 to 95. [29] This discrepancy arises from differing interpretations of usability, defined as the extent to which a system can be used effectively, efficiently, and satisfactorily by specific users to achieve predefined goals within a particular context. Usability testing evaluates the perspective of how well users can learn to operate a system or product to accomplish their objectives with satisfaction. [27].

Table 5 shows the mSUS score range and grade distribution for the Dents AR application ranging from grade F (poor) to grade A+ (excellent). Approximately 46.6% of responses fell within the “F&D” grade category or the poor adjective category, and approximately 53.3% of responses fell within the “C to A+” grade category or the OK to excellent adjective rating. These results differ with Nurazizah’s study, which reported that 81.27% achieved an A (Excellent) grade. [45] The difference may be attributed to variations in user demographics; Nurazizah’s [45] participants were teenagers and young adults in urban areas.

This result shows that exposure to digital technology shapes perceptions of usability. It further highlights the importance of introducing technology use at an early age in rural areas to enhance adaptability to new technologies for oral health promotion. These findings present important considerations for researchers and developers in designing the Dents AR system to be effective, efficient, and capable of achieving high satisfaction.

The limitations of this study highlight the need to gather additional information on smartphone ownership, usage duration, internet access, and signal strength, particularly in rural regions where the target population resides. Other influencing factors include device dependency, the potential novelty effect of first time AR use, and greater digital barriers faced by rural children. Another limitation is that usability findings may not generalize to devices with weaker cameras or processors and digital literacy, prior AR exposure, and smartphone usage patterns are not reported.

Another limitation of this study is that potential interfering factors were not examined. Although mSUS is a standardized, validated and widely used instrument appropriate for measuring the usability of the Dents AR application, it needs to be evaluated with other evaluation instruments to assess broader user experience such as enjoyment, emotional response, engagement or workload.

## Conclusion

The perspectives of a broader user population on the dents ar augmented reality application for children’s oral health promotion based on gender, age, and grade in rural areas, show that the Dents AR application has values ranging from grade F (poor) to grade A+ (excellent). Approximately 46.6% of responses fell within the “F&D” grade category or poor adjective category, while 53.3% of responses fell within the “C to A+” grade category or the OK to excellent adjective rating, and perspectives across gender, age, and grade revealed no usability differences. The implication of this research is that the perspectives of children in rural areas on the Dents AR augmented reality application, based on gender, age, and grade using the modified System Usability Scale, represent one stage in validating this instrument for oral health promotion in rural elementary school children.

## Data Availability

The datasets used and/or analysed during the current study are available from the corresponding author on reasonable request.

## Abbreviations

AR: Augmented Reality
mSUS: Modified System Usability Scale
